# Towards Determination of Distances Between Nanoparticles and Grafted Paramagnetic Ions by NMR Relaxation

**DOI:** 10.1007/s00723-017-0952-3

**Published:** 2017-10-05

**Authors:** A. M. Panich, N. A. Sergeev

**Affiliations:** 1 Department of Physics, Ben-Gurion University of the Negev, P. O. Box 653, 8410501 Be’er Sheva, Israel; 2 Department of Mathematics and Physics, Institute of Physics, University of Szczecin, 70-451 Szczecin, Poland

## Abstract

We developed an approach for determining distances between carbon nanoparticles and grafted paramagnetic ions and molecules by means of nuclear spin–lattice relaxation data. The approach was applied to copper-, cobalt- and gadolinium-grafted nanodiamonds, iron-grafted graphenes, manganese-grafted graphene oxide and activated carbon fibers that adsorb paramagnetic oxygen molecules. Our findings show that the aforementioned distances vary in the range of 2.7–5.4 Å and that the fixation of paramagnetic ions to nanoparticles is most likely implemented by means of the surface functional groups. The nuclear magnetic resonance data data are compared with the results of electron paramagnetic resonance measurements and density functional theory calculations.

## Introduction

In the last decades, low-dimensional carbon nanomaterials such as fullerenes, nanotubes, nanodiamonds, onions and graphene have attracted significant attention from the scientific community due to their unique electronic, optical, thermal, mechanical and chemical properties. High biocompatibility and low toxicity of nanodiamonds assume many eventual applications in the biomedical field, including biosensors, drug and vaccine delivery, cancer therapeutics, fluorescence probes and biomarkers for medical imaging [1–5]. Nanodiamonds are important physical systems for various nanotechnologies such as quantum computing and metrology, information processing and communications [6, 7]. Graphene and its derivatives are promising candidates for applications as materials for the next-generation nanoelectronic devices, energy-storage and paper-like materials, nanocapacitors, sensors and mechanical resonators [8, 9]. In addition to the fascinating intrinsic characteristics inherent in nanodiamond and graphene, their electronic and magnetic properties can also be controlled and modified by grafting different atoms and molecules to the surface. Particularly grafting of transition and rare-earth metal ions to the surface of these nanoparticles promises a great potential in a variety of applications from catalysis to spintronics, nanomagnetic devices and magnetic resonance imaging (MRI) [10–26]. The latter item means a paramagnetic metal complex with several coordination sites available for water molecules to interact with the unpaired electrons of the paramagnetic ion, which results in a reduction of the proton spin–lattice relaxation time and thus in a relatively high water proton relaxivity of this complex in aqueous solution. Such compounds are aimed to be used as MRI contrast agents.

To perform the aforementioned applications, one must first of all (i) establish the fact of ion grafting onto the surface of carbon nanoparticle (CNP) and (ii) conduct a detailed characterization of the CNP—metal complex using physical methods that are sensitive to the electronic structure and chemical bonding. Among a number of techniques, nuclear magnetic resonance (NMR) is well suited to solve these problems [18]. Since NMR spectroscopy probes local magnetic effects, solid-state NMR spectra are usually not sensitive to a small amount of paramagnetic impurities except for a slight line broadening. Herewith paramagnetic ions, being grafted to the CNP surface, strongly affect nuclear spin–lattice relaxation. This fact is usually used in the NMR studies of paramagnetic complexes in solutions [27]. In the present paper, we developed an approach for determining distances between carbon nanoparticles of different geometry and grafted paramagnetic ions and molecules using the data of solid-state nuclear spin–lattice relaxation. The approach is applied to Cu-, Co- and Gd-grafted nanodiamonds, Fe-grafted graphenes, Mn-grafted graphene oxide and activated carbon fibers that adsorb paramagnetic oxygen molecules. Our findings show that the fixation of paramagnetic ions to nanoparticles is most likely implemented via surface functional groups. The NMR data are compared with the results of electron paramagnetic resonance (EPR) measurements and density functional theory (DFT) calculations.

## Experimental Details

Highly purified detonation nanodiamonds (DND) with average diameter of 5 nm have been grafted by copper, cobalt and gadolinium ions (thereafter Cu-, Co- and Gd-DND); the ion grafting technique is described elsewhere [11–15]. Two iron-grafted graphene samples of different size, nano- and micrographene, have been synthesized in the laboratory of Prof. Huixin He at the Rutgers University using the microwave enabled eco-friendly technique [21]. According to the atomic force microscopy (AFM) data, the average lateral size of the nanographene (NGr) sheets is 13.1 nm and the average height is 0.83 nm, indicating predominantly mono- and bilayer graphene structures. The average lateral size and height of the microsized graphene sheets (LGr) are 1300 and 1.8 nm, respectively, indicating a few-layer graphene. Graphene oxide sample was prepared in the laboratory of Prof. Ruoff at the University of Texas at Austin using Hummers method through oxidation of graphite with KMnO_4_/H_2_SO_4_ [28]. Then it was dispersed into single-layer graphene oxide by sonication in water and, after filtration and drying under 10^−2^ Torr vacuum, the graphene oxide (GO) was obtained as a powder [25]. We have shown that the compound synthesized in this way reveals isolated Mn^2+^ ions, which originate from potassium permanganate used in the process of the sample preparation and are anchored to the graphene oxide planes [25, 26]. The average lateral size and average height of the obtained sheets are around 560 and 1.1 nm, respectively. Activated carbon fibers (ACF) FR-20 (Kuraray Chemical) comprise a three-dimensional network of nanographene domains; each domain is formed by a stacking of 3–4 graphene layers with the in-plane size of ~ 3 nm [29]. The out-gassed (oxygen-free) ACF sample was prepared by evacuation of the as-prepared one down to 10^−6^ mbar during 24 h at room temperature and subsequent sealing in a glass tube [30, 31]. All investigated samples were with natural abundance of ^13^C isotope.

^13^C NMR spectra and spin–lattice relaxation times *T*_1_ have been measured at room temperature in an applied magnetic field of 8.0 T (resonance frequency 85.857 MHz). Magnetization recovery in measuring *T*_1_ was fitted by a stretched exponential

1 $$M( t) = M_{\infty } \left\{ {1 - \exp \left[ { - \left( {t/T_{1} } \right)^{\alpha } } \right]} \right\},$$

which a is characteristic of the spin–lattice relaxation via paramagnetic defects and impurities [11–13, 15, 18, 21, 25, 26]. Here *M*_*∞*_ is the equilibrium magnetization, and the parameter *α* varies in the range of 0.5 < *α* < 1. EPR and magnetic susceptibility measurements of the studied samples show two contributions coming from (i) the carbon-inherited paramagnetic defects (mainly dangling bonds with unpaired electron spins  and substitutional nitrogen paramagnetic P1 centers in DND and edge states in graphene) and (ii) grafted paramagnetic ions. The density of the former defects N_ci_ was found to be 6.3 × 10^19^ spins/g in DND [11–13, 15, 18], 1.1 × 10^18^ spin/g in NGr and 3.7 × 10^18^ spin/g in LGr [21], 1 × 10^18^ spin/g in GO [25] and 3 × 10^19^ spins/g in ACF [32]. Herewith the Cu-, Co- and Gd-grafted DND show 1.67 × 10^19^ spin/g of Cu^2+^ and Co^2+^ ions and 7.85 × 10^19^ spin/g of Gd^3+^ ions, respectively [11–13, 15, 18]. Iron-grafted nanographene and micrographene samples show 7.81 × 10^19^ and 1.18 × 10^19^ iron (Fe^2+^ and Fe^3+^) ions, respectively [21]. Graphene oxide sample reveals 5.5 × 10^18^ spin/g of isolated impurity Mn^2+^ ions joint to the graphene sheets [25]. ACF samples reveal 6 × 10^19^ spin/g oxygen molecules attached to the particle surface [31, 32]. The data about the nanoparticle size and densities of the intrinsic paramagnetic defects and grafted ions and oxygen molecules are collected in Table 1.

**Table 1:** Average size of the studied nanocarbon particles and densities of carbon-inherited paramagnetic defects *N*_ci_ and of paramagnetic ions and oxygen molecules $N_{\text{S}}^{\text{PM}}$

Compound	Average diameter, nm	Average height, nm	*N*_ci_, spin/g	$N_{\text{S}}^{\text{PM}} ,$ spin/g
Cu-DND	5	–	6.3 × 10^19^	1.67 × 10^19^
Co-DND	5	–	6.3 × 10^19^	1.67 × 10^19^
Gd-DND	5	–	6.3 × 10^19^	7.85 × 10^19^
Mn-GO	560	1.1	1 × 10^18^	5.5 × 10^18^
Fe-NGr	13.1	0.83	3.7 × 10^18^	7.81 × 10^19^
Fe-LGr	1300	1.8	1.1 × 10^18^	1.18 × 10^19^
ACF	3	1.2	3 × 10^19^	6 × 10^19^

## Theory

Grafting of the transition and rare-earth ions to the surface of CNP has been proved by our ^13^C NMR spin–lattice relaxation measurements. The matter is that if the paramagnetic ions are bound to the CNP surface, the interaction of uncoupled electron spins of these ions with carbon nuclear spins opens an additional relaxation channel and thus results in noticeable reduction of the ^13^C spin–lattice relaxation time. This effect was observed in our experiments [11–13, 15, 18, 21, 25, 26, 31].

Spin–lattice relaxation rate $R_{1} = \frac{1}{{T_{1} }}$ of a nuclear spin *I* that interacts with unpaired electron spin *S* of a paramagnetic defect is given by the expression [33, 34]

2 $$R_{1} (r_{ik} ) = \frac{1}{{T_{n} \left( {r_{ik} } \right)}} = \frac{2}{15}\gamma_{S}^{2} \gamma_{I}^{2} \hbar^{2} S\left( {S + 1} \right)\left[ {\frac{{3\tau_{e} }}{{1 + \omega_{I}^{2} \tau_{e}^{2} }} + \frac{{7\tau_{e} }}{{\left( {1 + \omega_{e}^{2} \tau_{e}^{2} } \right)}}} \right]\left( {\frac{1}{{d_{ik}^{6} }}} \right)$$

Here *γ*_*I*_ and *γ*_*S*_ are the nuclear and electron gyromagnetic factors, *ω*_*I*_ = 2*π* *γ*_*I*_ *B*_0_ = 5.38 × 10^8^ s^−1^ and *ω*_*e*_ = 1.41 × 10^12^ s^−1^ are the ^13^C and electron Larmor angular frequencies in the applied magnetic field *B*_0_ = 8 T used in our experiment, respectively, $d_{ik}$ is the distance from the *i*th nucleus to *k*th paramagnetic center, and* τ*_*e*_ is the correlation time of the electron spin of paramagnetic ion.

Equation (2) shows that if magnetic inclusions are contained in a material as a separate phase, their effect on relaxation is negligible owing to the inversed sixth-power dependence of *R*_1_ on *d*. Herewith the paramagnetic ions bound to the CNP surface significantly accelerate nuclear spin–lattice relaxation. This effect allows determination the distance between the nanoparticle surface and grafted ions.

For a magnetically diluted system, in which the density of paramagnetic defects is of several orders of magnitude smaller than that of nuclei, nuclear spin–lattice relaxation rate *R*_1_ is proportional to the paramagnetic ion concentration [33, 34]. Suggesting that all paramagnetic ions are positioned at the same distance from the CNP surface, one can estimate the spacing between the ions and surface from the ^13^C spin–lattice relaxation data.

**Fig. 1:**
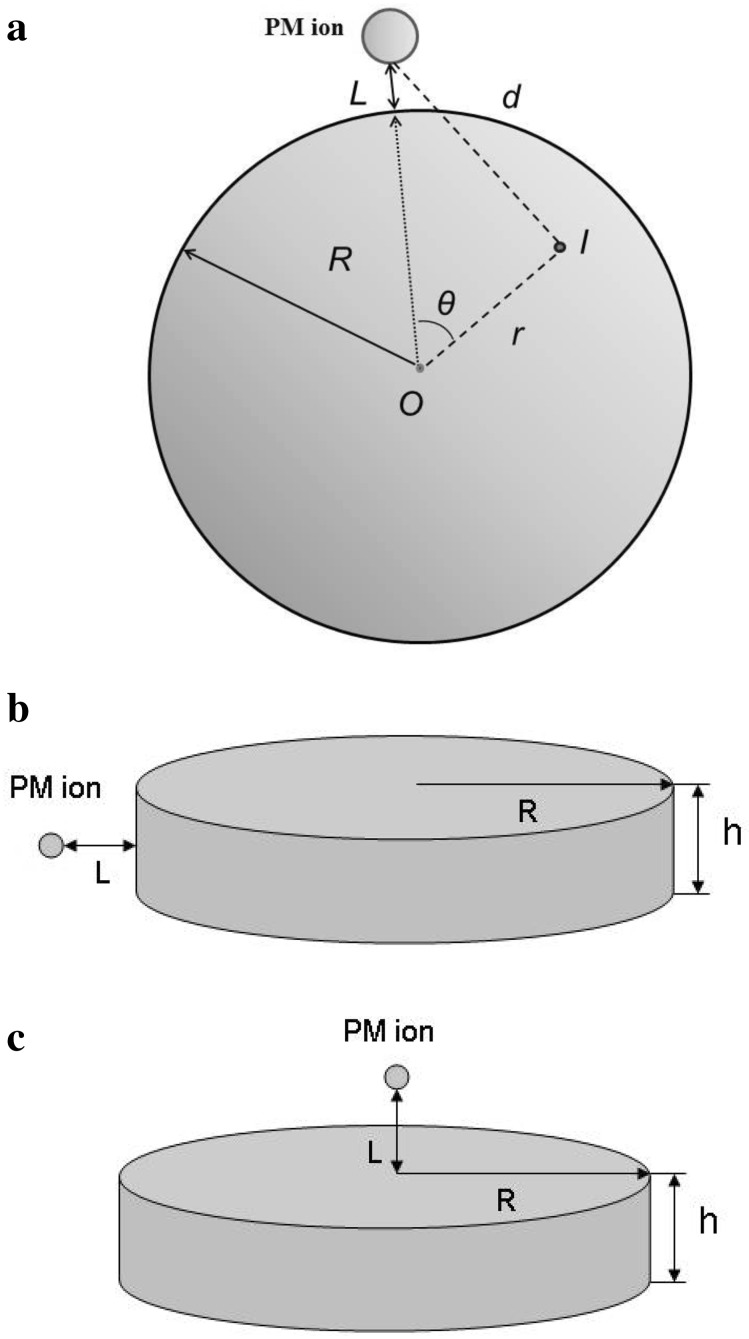
Sketches of the paramagnetic ion grafting to the spherical (**a**) and flat (disk-like) nanoparticles (**b**, **c**)

Let us first discuss an ion grafted to a spherical nanoparticle as shown in Fig. 1a, where *R* is the radius of spherical particle, *L* is the distance between the paramagnetic ion and its surface, *r* = OI is the distance between a nuclear spin *I* and the center of the ball, *d* is the distance between nuclear spin *I* and paramagnetic ion, and *θ* is the angle between the ion-O and OI directions. Note that we discuss the case when a magnetic ion is grafted to the nanodiamond surface from outside but not from inside (Fig. 1). To find the contribution of the paramagnetic ions to ^13^C nuclear spin–lattice relaxation, one should use Eq. (2) and calculate the integral (in spherical coordinates)

3 $$\frac{1}{{T_{1n}^{\rm PM} }} = R_{1n}^{\rm PM} = \frac{{{\rm CN}_{S}^{\rm PM} }}{V}\int\limits_{0}^{R} {\frac{{r^{2} {\rm d}r}}{{d^{6} }}} \int\limits_{0}^{\pi } {\sin \theta\, {\rm d}\theta \int\limits_{0}^{2\pi } {{\rm d}\phi} }$$

where

4 $$C = \frac{2}{15}\left( {\hbar \gamma_{I} \gamma_{S} } \right)^{2} S\left( {S + 1} \right)\left[ {\frac{{3\tau_{e} }}{{1 + \omega_{I}^{2} \tau_{e}^{2} }} + \frac{{7\tau_{e} }}{{\left( {1 + \omega_{e}^{2} \tau_{e}^{2} } \right)}}} \right],$$

$V = \frac{{4\pi R^{3} }}{3}$ is the volume of the particle, and $N_{\rm S}^{\text{PM}}$ is the number of the paramagnetic ions grafted to the surface of the particle. Correlation time *τ*_*e*_ of the electron spin of paramagnetic ions usually varies in the range of 10^−9^–10^−11^ s [35–38]. Therefore, (*ω*_e_ *τ*_e_)^2^ ≫ 1 and the second term in Eq. (4) containing the electronic Larmor precession frequency can be neglected compared with the first term. Using the law of cosines, Eq. (3) may be written as

5 $$\frac{1}{{T_{1n}^{\rm PM} }} = R_{1n}^{PM} = \frac{{{\rm CN}_{S}^{\rm PM} }}{V}\int\limits_{0}^{R} {r^{2} {\rm d}r} \int\limits_{0}^{\pi } {\frac{\sin \theta\, {\rm d}\theta }{{\left( {r^{2} + \left( {R + L} \right)^{2} - 2r\left( {R + L} \right)\cos \theta } \right)^{3} }}\int\limits_{0}^{2\pi } {{\rm d}\phi } } = \frac{{{\rm CN}_{S}^{\rm PM} }}{{\left[ {L\left( {2R + L} \right)} \right]^{3} }},$$

and thus

6 $$L = - R + \sqrt {R^{2} + \sqrt[3]{{\frac{{{\rm CN}_{S}^{\rm PM} }}{{R_{1} }}}}}$$

Let us now move to the plane particles such as graphene, graphene oxide and ACF. Two models of such particles, with different paramagnetic ions bound (i) to the particle edge or (ii) to its basal plane, are discussed in the literature. Using the aforementioned approach, one can derive the expressions for the distances between paramagnetic ions and the plane (disk-like) nanoparticles to which these ions are grafted. For the ion positioned at the distance *L* from the edge of the disk-like particle of radius *R* and thickness *h* (Fig. 1b), one should calculate integral

7 $$T_{1}^{ - 1} = R_{1} = \frac{{{\rm CN}_{S}^{\rm PM} }}{V}\iiint\limits_{V} {\frac{1}{{d^{6} }}}\,{\rm d}V \approx \frac{{{\rm CN}_{S}^{\rm PM} h}}{V}\int\limits_{0}^{R} {\rho {\rm d}\rho } \int\limits_{0}^{2\pi } {\frac{1}{{\left[ {\rho^{2} + \left( {R + L} \right)^{2} - 2\rho \left( {R + L} \right)\cos \phi } \right]^{3} }}} \,{\rm d}\phi ,$$

where *d* is the distance between the nucleus and paramagnetic ion and *ρ* is the distance from the nucleus to the center of the particle. Integration is done in cylindrical coordinates. Taking into account that $V = \pi R^{2} h$ the calculation yields

8 $$T_{1}^{ - 1} = R_{1} = {\rm CN}_{S}^{\rm PM} \frac{{\left( {R^{2} + 2\left( {R + L} \right)^{2} } \right)}}{{2L^{4} \left( {2R + L} \right)^{4} }}$$

Since usually in-plane particle size $R \gg L$, Eq. (8) can be transformed to

9 $$L = \sqrt[4]{{\frac{{3{\rm CN}_{S}^{\rm PM} }}{{32R^{2} R_{1} }}}}$$

When ion is grafted to the basal plane of graphene (Fig. 1c), one should calculate the integral

10 $$\frac{1}{{T_{1n}^{\rm PM} }} = R_{1n}^{\rm PM} = \frac{{CN_{S}^{\rm PM} }}{V}\int\limits_{0}^{2\pi } {{\rm d}\phi } \int\limits_{0}^{h} {{\rm d}z} \int\limits_{0}^{R} {\frac{r}{{\left( {r^{2} + \left( {L + z} \right)^{2} } \right)^{3} }}} \,{\rm d}r$$

in which *r* is the distance between the nucleus *I* and paramagnetic ion and *θ* is the angle between the *x*-axis and projection of ion—spin *I* vector to the *xy*-plane in the cylindrical coordinate system. The parameters *R*, *L*, *h* and *V* are given above. The calculation yields

11 $$\begin{aligned}R_{1n}^{\text{PM}} &= \frac{{{\text{CN}}_S^{\text{PM}}}}{V}\left\{ \frac{1}{{12{L^3}}} - \frac{1}{{12{{\left( {L + h} \right)}^3}}} + \frac{l}{{8{R^2}\left( {{R^2} + {L^2}} \right)}} \right. \\ &\quad\quad\left. - \frac{(L + h)}{{8{R^2}\left[ {{R^2} + {{\left( {L + h} \right)}^2}} \right]}} - \frac{{{\text{ arctg}}\left( {\frac{Rh}{{{R^2} + L\left( {L + h} \right)}}} \right)}}{{8{R^3}}} \right\}.\end{aligned}$$

Suggesting that the planar particle size is much larger than its thickness and than its distance to the ion, i.e. that *R* ≫ (*L* + *h*), Eq. (11) may be simplified to

12 $$R_{1n}^{\rm PM} \cong \frac{{{\rm CN}_{S}^{\rm PM} }}{{6R^{2} L^{3} h}}\left[ {1 - \frac{1}{{\left( {1 + \frac{h}{L}} \right)^{3} }}} \right].$$

If we consider one- or few-layer particles, in which *h* ~ *L*,

13 $$L \cong \sqrt[3]{{\frac{{{\rm CN}_{S}^{\rm PM} }}{{6R^{2} hR_{1} }}}}$$

In the next section, the received formulas will be used to determine the ion-particle separations.

## Experimental Results and Calculation of Ion-Particle Distances From Nuclear Spin–Lattice Relaxation Data

**Fig. 2:**
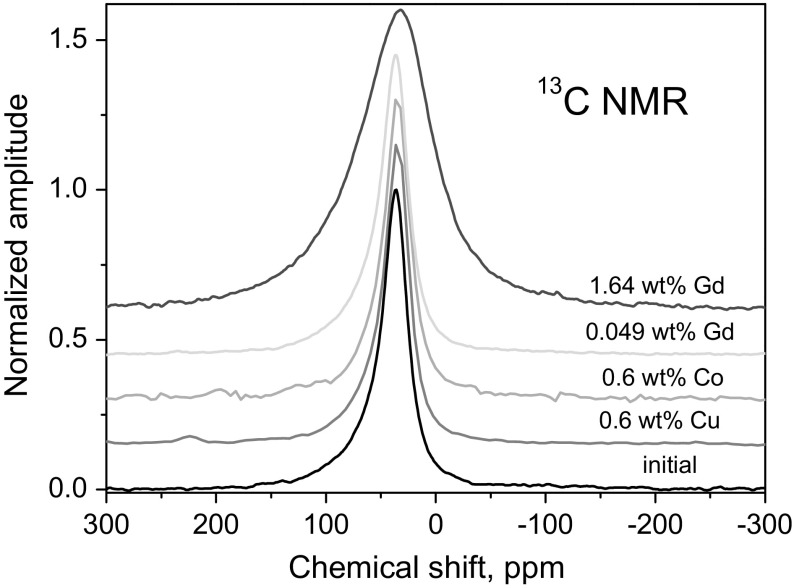
Characteristic ^13^C NMR spectra of initial DND and those grafted by different paramagnetic ions

Typical ^13^C spectra of initial nanodiamond sample and those grafted by paramagnetic ions [11–13, 15, 18] are shown in Fig. 2. As it was mentioned above, solid-state NMR spectra are usually not sensitive to a small amount of paramagnetic impurities except for a slight line broadening. Thus, while Gd-DND with large magnetic moment of Gd^3+^ ion (*S* = 7/2) reveals a gradual broadening of the ^13^C line with an increase in the Gd concentration [15], such effect is not observed in DND grafted by Cu^2+^ and Co^2+^ ions with *S* = 1/2. Similar absence of the line broadening was obtained also in the graphene and ACF samples under study [21, 25, 26, 31].

Anyhow, a broadening of the NMR line does yet not evidence ion grafting, since this effect can also be caused by separately located magnetic inclusions.

**Fig. 3:**
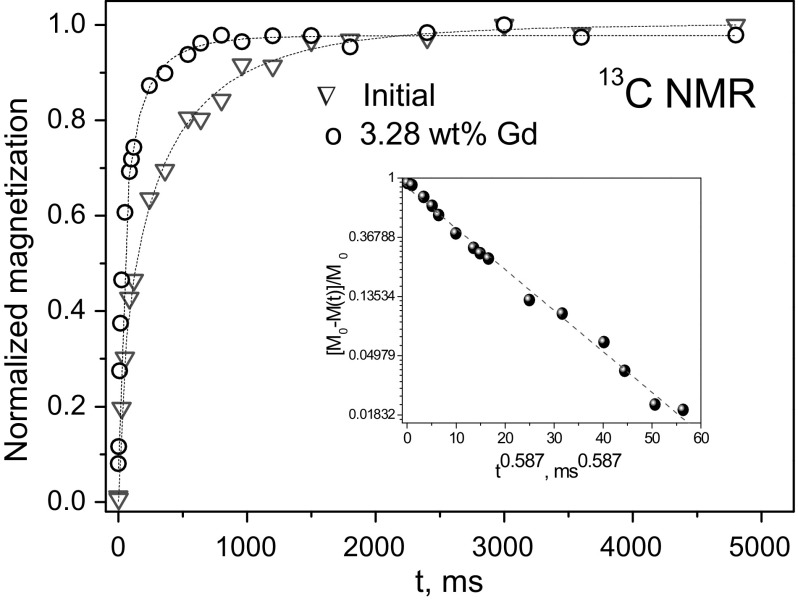
Magnetization recovery (*T*_1_ measurements) for initial DND and Gd-DND samples corresponding to *T*_1_ = 262 and 89 ms, respectively. Dashed lines are simulations using Eq. (1). Inset shows stretched exponential ^13^C magnetization recovery of the diamond core carbons on a semi-logarithmic scale

Herewith, a noticeable acceleration of the nuclear spin–lattice relaxation after the modification of the particles’ surface by transition and rare-earth ions has been obtained [11–13, 15, 18, 21, 25, 26]. It is well seen from the characteristic magnetization recovery curves of initial and Gd-grafted DND (Fig. 3). These findings unambiguously show grafting of the aforementioned carbon nanoparticles by the paramagnetic ions. This conclusion is also supported by the experimentally obtained linear increase in the relaxation rate with increased paramagnetic ion concentration [11–13, 15, 18, 21, 25, 26]. Similar relaxation effects, caused by paramagnetic iron and manganese ions, were obtained in graphene and graphene oxide [21, 25, 26]; that caused by oxygen molecules was observed in activated carbon fibers, ACF, by means of comparable measurements of as-received samples and those evacuated down to 10^−6^ mbar samples [31]. The only reason for the obtained *R*_1_’s increase is the interaction of the ^13^C nuclear spins with the electron spins of the grafted paramagnetic ions and molecules. Taking into account the proportionality of *R*_1_(^13^C) to the inversed sixth power of the distance between the nucleus and paramagnetic ion/molecule [see Eq. (1)], such mechanism is effective only in the case that paramagnetic ions are attached to the CNP surface rather than existing as a separate phase in the compound. It allows us to conclude that the paramagnetic copper, cobalt, gadolinium, manganese and iron ions and the oxygen molecules are anchored to the particle surface and presumably form charge-transfer complexes. Herewith, the spin–lattice relaxation rate caused by paramagnetic ions and molecules is [11, 13, 15, 18, 31]

14 $$R_{1}^{\rm PM} = \frac{1}{{T_{1}^{\rm PM} }} = \frac{1}{{T_{1}^{\exp } }} - \frac{1}{{T_{1}^{ci} }},$$

where $T_{1}^{\exp }$ is the experimental value of *T*_1_ and $T_{1}^{ci}$ is the relaxation time in the initial (non-grafted) sample, in which the relaxation is caused by interaction of ^13^C nuclear spins with the carbon-inherited paramagnetic centers.

The number of paramagnetic ions and oxygen molecules per particle *N*_S_, determined by SQUID and EPR measurements [11–13, 15, 18–21, 25], as well as the correlation times *τ*_e_ of the electron spins of these ions and molecules [35–43] are given in Table 2.

**Table 2:** Electron correlation times *τ*_e_ and number of grafted paramagnetic ions and oxygen molecules per particle *N*_S_, relaxation rates caused by the interaction of ^13^C nuclear spins with grafted paramagnetic ions and molecules $R_{1}^{\text{PM}} ,$ and distance *L* between paramagnetic ions and molecules and nanoparticle surface

Compound	PM agent	*τ*_e_, *s*	*N*_S_	$R_{1}^{\rm PM}$, s^−1^	*L*, Å	Grafting mode
Cu-DND	Cu^2+^	10^−8^–10^−10^	4	1.06	3.2 ± 0.4	
Co-DND	Co^2+^	10^−9^–10^−10^	4	0.801	3.6 ± 0.4	
Gd-DND	Gd^3+^	1 × 10^−11^–3 × 10^−11^	18	7.34392	3.1 ± 0.3	
Fe-NGr	Fe^2+^/Fe^3+^	10^−11^–10^−13^	16	0.184	3.5 ± 1.1	Edge
Fe-LGr	Fe^2+^/Fe^3+^	10^−11^–10^−13^	47,000	0.04	3.7 ± 1.2	Edge
Fe-NGr	Fe^2+^/Fe^3+^	10^−11^–10^−13^	16	0.184	3.2 ± 1.3	Plane
Fe-LGr	Fe^2+^/Fe^3+^	10^−11^–10^−13^	47,000	0.04	2.7 ± 1.1	Plane
Graphene oxide	Mn^2+^	5 × 10^−11^–2 × 10^−12^	2675	0.018	5.4 ± 1.1	Edge
Graphene oxide	Mn^2+^	5 × 10^−11^–2 × 10^−12^	2675	0.018	5.2 ± 1.5	Plane
ACF	O_2_	10^−11^–10^−13^	0.9	0.141	3.5 ± 1.0	Edge
ACF	O_2_	10^−11^–10^−13^	0.9	0.141	2.9 ± 1.0	Plane

**Fig. 4:**
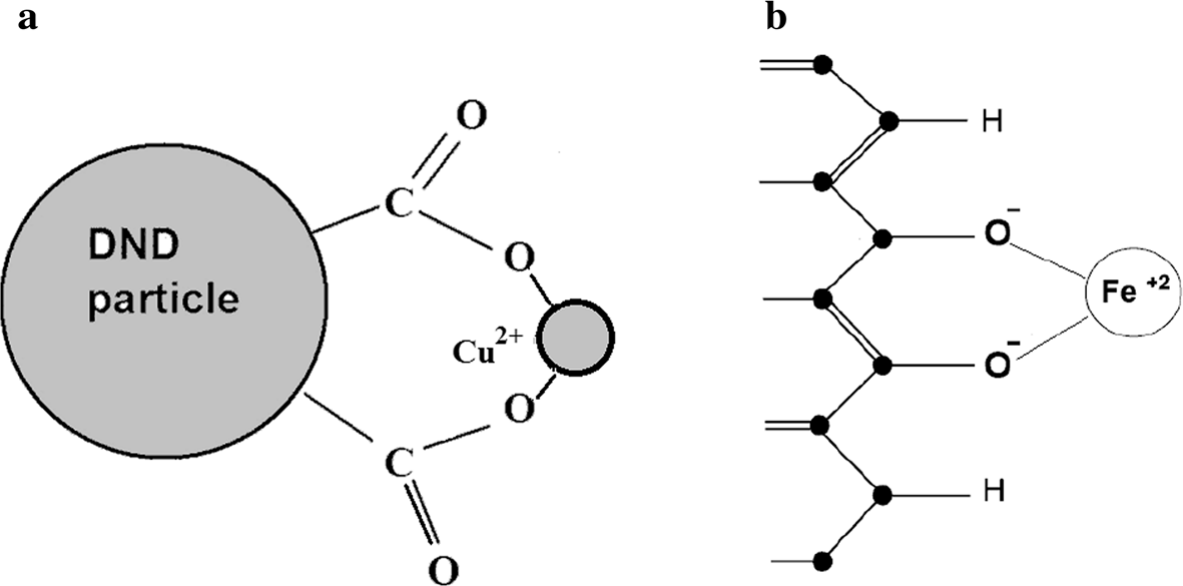
Sketch of (**a**) Cu^2+^ ion fixation to the nanodiamond surface and (**b**) Fe^2+^ ion fixation to the graphene edge via a pair of neighboring deprotonated carboxyl groups. Black spheres in (**b**) are carbon atoms

Let us start with paramagnetic ions grafted to the nanodiamonds. The aforementioned model (Fig. 1a) is based on a spherical approximation of particles, though the DND particle shapes may be somewhat irregular. However, we deal with powder DND samples, and our initial expression Eq. (1) already involves angular averaging of anisotropic electron-nuclear interactions. This fact additionally smoothes the significance of the particles form factor making it to be not crucial. The calculation using the nuclear spin–lattice relaxation data obtained (Table 2) and Eq. (5) yields the distance *L* between the paramagnetic ion and DND surface in the range from 3.1 to 3.6 Å (Table 2). Such distances are expected for C-COO-Gd (Cu, Co) fragments with typical C–C and C=O bond lengths of 1.54 and 1.29 Å, Gd–O, Co–O and Cu–O bond lengths of 2.4, 2.1 and 1.9 Å, respectively, and O=C–O angle of ~ 120° in carboxyl group, meaning that Gd^3+^, Cu^2+^ and Co^2+^ ions substitute hydrogen atoms in the COOH groups as proposed in the previous studies [11–15, 18, 19]. This finding is in agreement with that estimated by the analysis of the broadening of the EPR line of carbon-inherited defects located in the DND core caused by Gd grafting, which shows that the distance between the Gd ion and the DND surface should not exceed 4 Å [19]. This also agrees well with the analysis of the EPR data of Cu-DND [19], which shows that the most probable distance of Cu^2+^ ions from the plane of uppermost carbon atoms of the (111) diamond surface should be recognized as 3.1 Å. A realistic sketch of the Cu^2+^ ion positioning on the nanodiamond surface terminated by oxygen-containing groups is shown in Fig. 4a. This model was constructed similar to those of copper–DND binding studied by the DFT method [44], which examines coordination of the metal cation by different surface groups. The water molecules surrounding the gadolinium ion provide additional coordination of the ion. Preliminary results of DFT calculations of this complex yield a lower limit of the Gd-DND distance as 2.74 Å [15], which corresponds well to the value found from the analysis of the NMR relaxation data.

Let us now move to the grafted graphene and graphene oxide nanoparticles sketched in Fig. 1b, c. Calculations predict several models of ion anchoring to graphene. Wu et al. [45] reported on the Mn atom substituting a carbon atom or adsorbed on a vacancy site in the graphene sheet and bound with three neighbor carbon atoms, forming a charge-transfer complex with covalent Mn–C bonds. AlZahrani [46] calculated that the Mn atom is not likely to substitute the C atom but prefers to be interstitially adsorbed at the center of the hexagon, forming covalent bonds with the nearest carbon atoms. Next, some chemically derived graphene monolayers comprise defect-free graphene areas interspersed with defect areas dominated by clustered pentagons and heptagons [47]. The latter defects are also available for paramagnetic ions. First-principle calculations [48] predict that 3*d-*transition metal atoms exhibit a covalent bonding with graphene due to hybridization between the *d*_*x*2−*y*2_ and *d*_*yz*_ orbitals of the metal atoms and *p*_*z*_ orbitals of the carbon atoms. All these models predict occurrence of charge-transfer complexes between ions and graphene. Such predictions correlate well with our experimental data, which show that the impurity ions are grafted to the graphene and graphene oxide layers [21, 25, 26]. However, the way of grafting seems to be different.

In our study, we calculated the ion–nanoparticle distances for both edge (Fig. 1b) and plane (Fig. 1c) ion grafting. The results of the calculations are collected in Table 2. One can find that the edge and plane iron attachments to the nano- and micrographene particles yield similar ion-graphene separations varying in the range from 2.7 to 3.7 Å. These separations are longer than the length of covalent bond between the ion and nearest carbon atom. Therefore, along with our recent study of Fe-grafted graphenes [21], we suggest that the fixation of ions to zigzag graphene edges is formed by means of functional groups, mostly of > C=O (Fig. 4b). Such kind of ion attachment is confirmed by reduction of the EPR signal coming from unpaired electron spins of the edge states after the iron grafting. Therefore, we are led to the conclusion that the grafting of Fe ions to the graphene edge is more realistic in the case in question. The calculated Fe-graphene distances are very reasonable taking into account typical C–O and Fe–O chemical bond lengths. At that, the Mn–GO distances obtained for the graphene oxide, 5.2–5.4 Å, seem to be somewhat longer than expected. This result may be explained as follows: Eq. 2 describes direct nuclear spin–lattice relaxation due to the dipole–dipole coupling between electron and nuclear spins. An additional relaxation mechanism is spin diffusion among nuclear spins, which allows spin magnetization of the distant nuclei to be spatially transferred to paramagnetic defects. Low natural abundance of ^13^C isotope (1.07%) makes carbon materials to be nuclear spin diluted, which, in turn, yields ^13^C nuclear spin diffusion to be quite slow. Detailed analysis of the spin diffusion in diamonds [49–51] shows that for the concentration of paramagnetic centers $N_{ci} < 6 \times 10^{18} \frac{\text{spin}}{g}$ all ^13^C nuclei, excluding those located inside the sphere of diffusion barrier radius, relax via spin diffusion. Increase in $N_{S}$ causes an increase in the number of ^13^C nuclei which relax directly and decrease of those that relax via spin diffusion. Above $N_{ci} = 4.2 \times 10^{19} \frac{\text{spin}}{g}$ all ^13^C nuclei are relaxed directly without spin diffusion. It means that the spin diffusion is suppressed in all nanodiamond samples with $N_{ci} = 6.3 \times 10^{19} \frac{\text{spin}}{g}$ (Table 1) studied in our paper, thus the use of Eq. 2 is correct. Next, our calculations done for planar systems lead to conclusion that ^13^C spins in Fe-NGr, Fe-LGr and ACF samples also reveal only direct relaxation according to Eq. 2, while spin diffusion may play some role in Mn–GO, in which the calculated distance between the paramagnetic centers (~ 41 Å) exceeds the doubled diffusion barrier radius (2 × 15.7 Å). Therefore, the use of Eq. 2 for this sample is somewhat limited, which can result in somewhat longer ion-to-surface distance calculated.

Let us now move to ACF, in which, as it was shown in Ref. [31], ^13^C nuclear spin–lattice relaxation is driven by two contributions, namely by interaction of nuclear spins with (i) carbon-inherited paramagnetic defects and (ii) oxygen molecules adsorbed onto the surface of these carbon nanoparticles. The ground state of molecular oxygen reveals two electrons with parallel spins in the highest occupied 2*p*π_g_* molecular orbital, leading to the paramagnetic moment of the molecule. The latter yields the oxygen-driven nuclear spin–lattice relaxation observed in ACF. Our calculations yield 3.5 Å for the edge attachment of oxygen molecules and 2.9 Å for the plane attachment.

The experimental error in measurements of the spin–lattice relaxation rate usually does not exceed 10%. Taking into account that the distance *L* between the nanoparticle and paramagnetic ion is proportional to the roots of third, fourth and sixth degrees of $R_{1}^{PM}$[Eqs. (6), (7) and (13)], the error in measurements of $R_{1}^{\rm PM}$ does not yield significant contribution to the uncertainty of the calculated value of *L*. The main reason of the uncertainty in *L*, given in Table 2, is due to the variations of the electron correlation time *τ*_e_ reported in the literature (see Table 2), which were taken into account in our calculations of *L*. Direct measurements of *τ*_e_ in the studied compounds would significantly increase the accuracy in the determination of *L*.

## Summary

We developed the approach for determining distances between carbon nanoparticles and grafted paramagnetic ions and molecules using the data of nuclear spin-lattice relaxation. The approach was successfully applied to copper-, cobalt- and gadolinium-grafted nanodiamonds, iron-grafted graphenes, manganese-grafted graphene oxide and activated carbon fibers that adsorb paramagnetic oxygen molecules. Our results show that the aforementioned distances vary in the range of 2.7–5.4 Å consistent with the fixation of paramagnetic ions to nanoparticles through the surface functional groups. The NMR data are compared with the results of EPR measurements and DFT calculations. The developed approach can be applied to different types of nanoparticles and grafted paramagnetic species.
